# Effects of fish oil supplementation on inflammatory acne

**DOI:** 10.1186/1476-511X-11-165

**Published:** 2012-12-03

**Authors:** Golandam Khayef, Julia Young, Bonny Burns-Whitmore, Thomas Spalding

**Affiliations:** 1 Department of Human Nutrition and Food Science, California State Polytechnic University, Pomona, California, USA

**Keywords:** Acne Vulgaris, Fish Oil, Inflammation, n-3 Fatty Acid, Omega 3 Fatty Acid, EPA

## Abstract

**Background:**

Given that acne is a rare condition in societies with higher consumption of omega-3 (n-3) relative to omega-6 (n-6) fatty acids, supplementation with n-3 may suppress inflammatory cytokine production and thereby reduce acne severity.

**Methods:**

13 individuals with inflammatory acne were given three grams of fish oil containing 930 mg of EPA to their unchanged diet and existing acne remedies for 12 weeks. Acne was assessed using an overall severity grading scale, total inflammatory lesion counts, and colorimetry.

**Findings:**

There was no significant change in acne grading and inflammatory counts at week 12 compared to baseline. However, there was a broad range of response to the intervention on an individual basis. The results showed that acne severity improved in 8 individuals, worsened in 4, and remained unchanged in 1. Interestingly, among the individuals who showed improvement, 7 were classified as having moderate to severe acne at baseline, while 3 of the 4 whose acne deteriorated were classified as having mild acne.

**Conclusion:**

There is some evidence that fish oil supplementation is associated with an improvement in overall acne severity, especially for individuals with moderate to severe acne. Divergent responses to fish oil in our pilot study indicates that dietary and supplemental lipids are worthy of further investigation in acne.

## Background

Acne vulgaris is a common yet complex inflammatory skin disease in Westernized nations and the overall occurrence rate appears to be rising [1,2]. Inflammatory mediators are predominantly released by activated leukocytes and result in inflammatory acne lesions characterized by pain, redness, and swelling [3]. Given that acne is a rare condition in non-Westernized societies with higher ratios of n-3 to n-6 from dietary intake, it appears that the lower n-3 content of the western diet is an important dietary modulator of these inflammatory mediators [1,4-6]. A case control study of Koreans found that individuals with acne consumed significantly less fish and more junk food than the control group [7]. A similar study of an Italian population found that consumption of fish was associated with a protective effect against moderate to severe acne [8]. This inverse association between fish consumption and acne severity is expected because fish contains high levels of n-3 fatty acid eicosapentaenoic acid (EPA) that acts as a competitive inhibitor of AA conversion to inflammatory mediators, PGE_2_ and LTB_4,_ which leads to reduced inflammatory acne lesions [9]. Some of these mediators include n-6 eicosanoids, prostaglandin E2 (PGE_2_), and leukotrine B4 (LTB_4_) that are derived from arachidonic acid (AA), an n-6 polyunsaturated fatty acid. Cytokines such as interleukin 1B (IL-1B) and tumor necrosis factor α (TNF-α) are also important inflammatory mediators [10]. It has also been shown that n-3 fatty acid supplementation suppresses the production of TNF-α and IL-Iβ in healthy individuals [3,10-12]. Despite the fact that the anti-inflammatory properties of EPA have been well-established in the literature, very few human studies have examined the clinical effects of this n-3 fatty acid on reducing inflammation in acne patients. A recent retrospective study examined the effect of a poly-nutrient supplementation containing EPA and antioxidants on 5 patients with mild to moderate acne who had consistently used the supplement for 2 months. Inflammatory acne lesion count was significantly reduced in all patients [13]. Given that the various studies that supplementation with antioxidants may help reduce severity of acne, it would be interesting to investigate whether fish oil can exert the same effects [14-17]. The objective of this study was to detect the isolated effects of EPA in the form of fish oil on severity of inflammatory acne in young healthy males.

## Methods

Healthy males ages 18–40 with mild to severe acne were recruited and screened to ensure they were not frequent consumers of n-3 fatty acids and have not received and are not currently receiving intense acne treatments. Each participant was required to consume 3 fish oil capsules daily for 12 weeks that contained a total of 930 mg EPA, 720 mg DHA, and 174 mg DPA per 3 capsules. Participants continued their usual diets but were required to complete 4 separate 3-day food diaries throughout the intervention to ensure they were not consuming large amounts of additional n-3 rich foods. They also logged the type of acne cleansers or medication (if any) that were taken daily. Compliance was measured also by participants’ self-reporting. Photos of the face were taken at baseline, week 6, and 12 with a Canon Rebel XS camera under the same temperature and lighting. Using these photos, total inflammatory lesions counts and acne severity grades were determined using the Allen and Smith grading scale (Table 1) [18]. In addition, objective measurements of skin redness were obtained using a Konica Minolta CR-400 colorimeter using the L*a*b* color system as defined by the Commission Internationale de L’Eclairage. One control of normal non-inflamed facial area and two or more acne areas were predetermined and marked on the photos for each participant. The same areas were measured at baseline, week 6 and 12, each measurement being an average of 3 repeats by 1 observer. Redness is signified by increasing positive values of a* above 0 and lightness is denoted by increasing values of L* from 0 to 100. We obtained the difference between the control and acne areas for each participant as Δ = acne – control.

**Table 1:** Changes in acne grade severity after 12 weeks of fish-oil supplementation

	**Acne Grade n (%)**				
	**0**	**2**	**4**	**6**	**8**
Before Treatment	1 (7)	4 (30)	2 (15)	2 (15)	4 (30)
After Treatment	2 (15)	5 (38)	2 (15)	3 (23)	1 (7)

A total of 16 healthy men were enrolled in this study and 13 completed the protocol. Two participants withdrew from the study and 1 participant was excluded from the data analysis due to self-reported compliance of less than 70%. The participants were young and had a BMI consistent with being normal in weight [19]. Seven participants (54%) identified their ethnicity as Hispanic/Latino, 3 as Caucasian (23%), and 3 (23%) as Asian.

The research protocol was approved by the Human Subjects Committee of the Institutional Review Board at California State Polytechnic University, Pomona, protocol # 10-190.

## Findings

**Figure 1:**
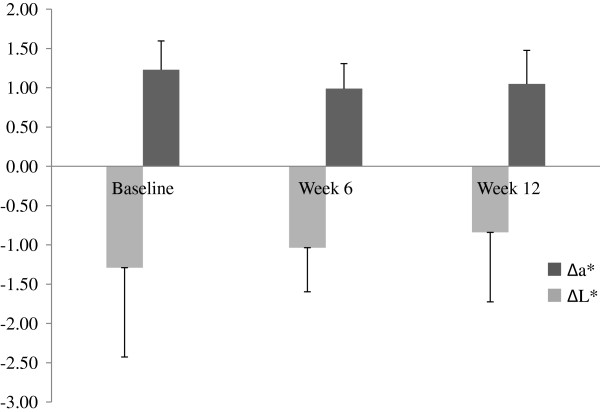
Changes in Δa* and ΔL* throughout 12 weeks of fish-oil supplementation.

A one-tailed Wilcoxon Signed Ranks test did not reveal a significant difference between Week 0 and Week 12 acne grade, *Z* (Week 12-Week 0) = −1.044, *p* > .05. A total of 8 (62%) participants improved at least one-point acne grade over the 12 weeks of supplementation. Acne grade worsened for 4 (31%) participants and did not change for 1 (7%) individual. Table 1 shows the number (and percentage) of individuals classified in each acne grade before and after supplementation. A one-tailed *t*-test for dependent means did not reveal a significant difference in mean acne counts for Week 0 and Week 12, *t*(12) = 0.338, *p* > .05. Mean acne count in Week 0 was 30 (*SE* = 5.06) and 31 (*SE* = 5.67) in Week 12. As expected, the unbiased effect size was small, *d*' = 0.08 (*SE*_d'_ = 0.26). One-way repeated measures analysis of variance did not reveal significant difference in mean Δa* or ΔL* across weeks *F(2,24) < 1.0*, Huynh-Feldt ε = 1.000, *p* > 0.05 (Figure 1).

**Figure 2:**
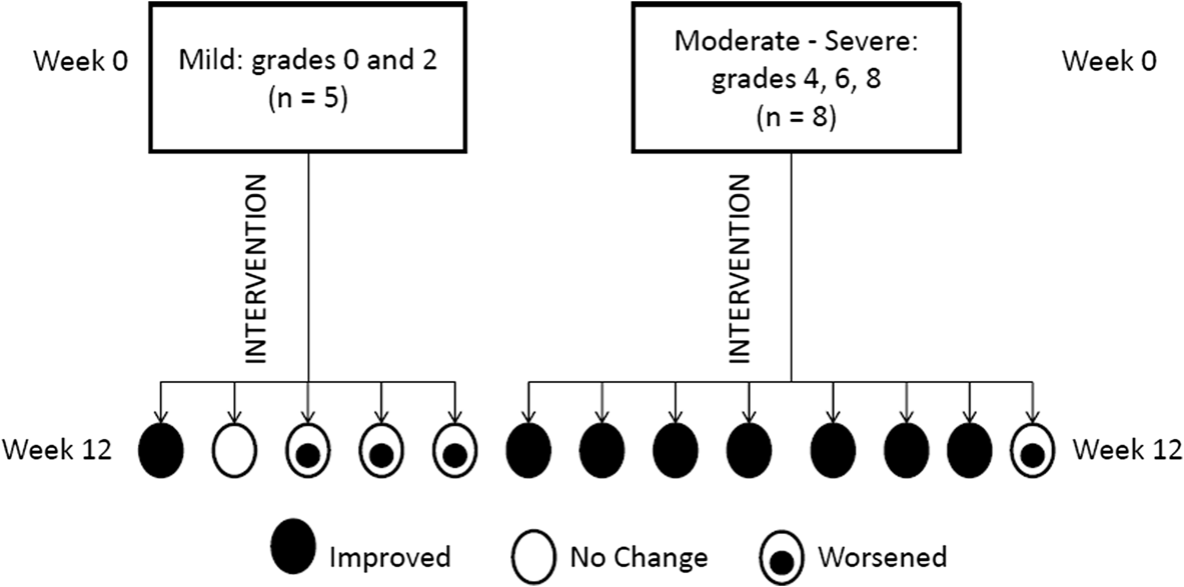
Difference in acne severity changes among individuals with mild vs. moderate-severe acne at baseline.

Even though statistical significance was not obtained for acne severity and lesion counts, the results of this study should be interpreted with caution and are likely inconclusive, for we clearly observed that the change in overall acne ratings over the 12-week supplementation period was in the predicted direction. After 12 weeks of supplementation, 8 out of 13 participants received a lower acne grade (i.e., indicating improvement) than at baseline. Five of these 8 individuals improved one grade and the rest improved at least two grades. One participant’s acne grade shifted from the most severe (grade 8) to the least severe grade (grade 0). Prior to supplementation, nearly one-third of the participants (n = 4) had an acne grade of 8, which is the most severe grade based on the scale used. All of these individuals had a less severe acne rating, as indicated by a lower grade at the end of the study. Of the 5 individuals who did not show improvement, acne severity worsened for 4 individuals and did not change for one individual. Notably, of the 4 individuals whose acne worsened, 3 individuals had mild acne (grades 0 or 2) at the start of the study (Figure 2). The individual whose acne did not change also exhibited mild acne (rating of 2) at the start of the study. On the other hand, 7 of the 8 individuals who entered the study with moderate to severe acne (grades 4, 6, and 8) saw improvement in acne severity by the end of the study. This may be the reason why significant results were not obtained in this study, at least for the acne severity ratings. The sample population included a relatively large proportion (38.5%) of individuals with mild acne. Since their acne grades were already near the low end of the scale at the start of the study, there was little room for obvious improvement and conversely greater opportunity for worsening. Similarly, a lack of significant difference in acne lesion counts between Weeks 0 and 12 may be partially due to a floor effect; that is, the inclusion of several individuals who had mild acne at the start of the study. These findings raise the question of whether the efficacy of fish oil supplementation is dependent on the initial acne severity. In other words, perhaps only individuals with moderate to severe acne can benefit from fish oil supplementation.

In addition, it should be noted that in counting the inflammatory lesions, we did not differentiate between the types of inflammatory lesions for our data analysis. In other words, we did not distinguish between papules, pustules, and cysts in the lesion count, but rather categorized them all as inflammatory lesions. This may be why we did not achieve high correlation between a* and actual inflammatory lesion counts, 0.42, 0.62, and 0.44 at baseline, week 6, and week 12, respectively. Thus, given the fact that acne is a dynamic disease, any improvement in the type of inflammatory lesion might have not been accounted for in our study. For the same reasons, more accurate and reliable results could be obtained if a larger measuring head were used to obtain the redness and lightness measurements.

Although non-significant, there was a visible decrease in mean Δa* at week 6 (Figure 1) which suggest that fish oil supplementation for 6 weeks is sufficient to reduce inflammation, because a* is well-understood to be correlated with erythema (increased blood flow to the skin) [20,21]. The non-significant linear increase in ΔL* from baseline to week 12 is also consistent with existing studies that found an inverse relationship between a* and L* in inflammatory skin diseases [22,23]. However, there should be more investigation into whether L* correlates with subjective assessment of different stages of an acne lesion.

Although we cannot draw any firm conclusions from our study with a small sample size and no placebo group, there is some promising evidence that fish oil supplementation is associated with an improvement in ratings of overall acne severity, especially for individuals with moderate and severe acne. It is possible that increasing the dose of EPA from 930 mg to 3–6 grams daily, as recommended for arthritis patients, would reveal more significant results [24]. In addition, effects of n-3 fatty acids should be examined in cohorts of subjects with the same acne severity grades or lesion counts in order to isolate the potential effect on different types of acne severity.

## Consent

**Figure 3:**
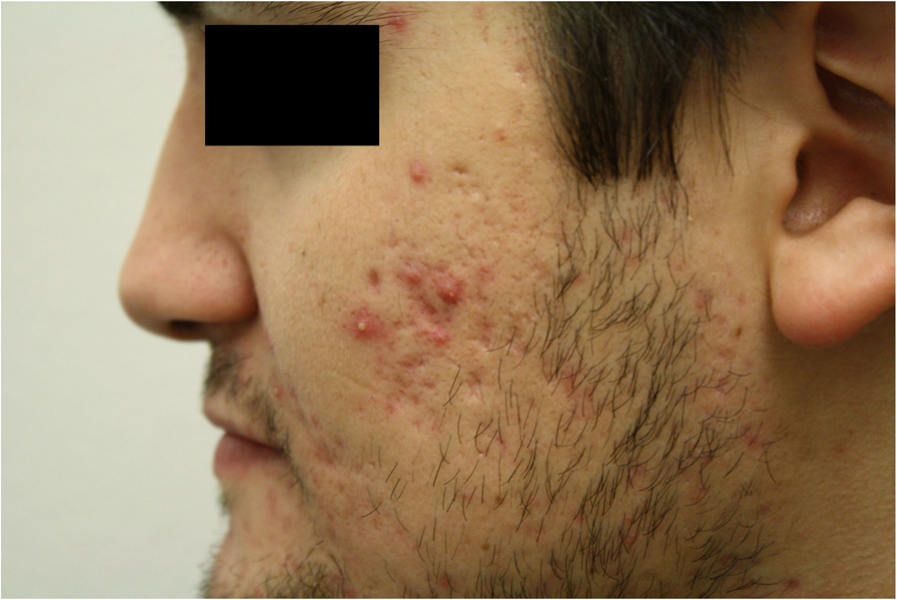
Participant photo before use of fish-oil supplements at baseline.

**Figure 4:**
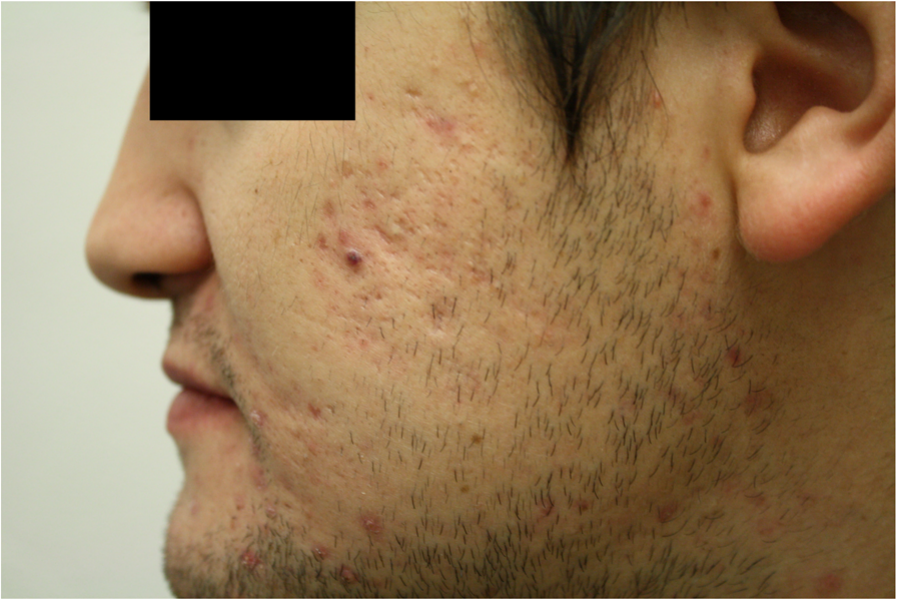
Participant photo after use of fish-oil supplements at week 12.

All of those participants in this study provided signed consent forms. The subject in Figures 3 and 4 provided consent for the use of his photographic images.

## Competing interests

The authors and corresponding authors of this study report no conflict.

## Authors’ contributions

GK and JY performed recruitment, screening, and data gathering. BBW and TS conducted statistical analyses and reviewed the manuscript. All authors read and approved the final manuscript.

## Authors’ information

GK and BBW are registered dietitians in the state of California.
